# The in vivo role of Rev1 in mutagenesis and carcinogenesis

**DOI:** 10.1186/s41021-020-0148-1

**Published:** 2020-02-28

**Authors:** Megumi Sasatani, Elena Karamfilova Zaharieva, Kenji Kamiya

**Affiliations:** Department of Experimental Oncology, Research Institute for Radiation Biology and Medicine, Hiroshima University, Hiroshima, 734-8553 Japan

**Keywords:** Translesion synthesis, Rev1, Mutagenesis, Tumorigenesis

## Abstract

Translesion synthesis (TLS) is an error-prone pathway required to overcome replication blockage by DNA damage. Aberrant activation of TLS has been suggested to play a role in tumorigenesis by promoting genetic mutations. However, the precise molecular mechanisms underlying TLS-mediated tumorigenesis in vivo remain unclear. Rev1 is a member of the Y family polymerases and plays a key role in the TLS pathway. Here we introduce the existing to date *Rev1*-mutated mouse models, including the *Rev1* transgenic (Tg) mouse model generated in our laboratory. We give an overview of the current knowledge on how different disruptions in *Rev1* functions impact mutagenesis and the suggested molecular mechanisms underlying these effects. We summarize the available data from ours and others’ in vivo studies on the role of Rev1 in the initiation and promotion of cancer, emphasizing how *Rev1*-mutated mouse models can be used as complementary tools for future research.

## Introduction

The genome of all living organisms is continually attacked by endogenous and exogenous genotoxic DNA damaging agents, such as metabolic processes, UV light, ionizing radiation, etc., and various DNA repair pathways have evolved to protect genome integrity. TLS is a mechanism of DNA damage tolerance involving specialized DNA polymerases with the capacity to replicate across damaged DNA template [1–5]. Mammalian TLS polymerases include Y-family polymerases (Pol η, Pol ι, Pol κ, and Rev1), 1 B-family polymerase (Pol ζ: Rev3L/ Rev7/ PolD2/ PolD3), 2 A-family polymerases (Pol θ and Pol ν), and three X-family polymerases (Pol μ, Pol λ, and Pol β) [6].

Rev1, in tandem with Pol ζ, stands out as a central player in error-prone TLS [3, 4, 7]. Rev1 is a deoxycytidyl transferase which incorporates deoxycytosines opposite structurally diverse damaged nucleotides, such as ^6^O-meG, a guanine with a large adduct at the C8 or N2 position, or abasic sites [8–17]. However, the most important function of Rev1 in error-prone TLS is regulatory rather than catalytic by recruiting the Y-family polymerases Pol η, Pol ι and Pol κ and the B-family Pol ζ, which interacts with Rev1 via its Rev7 component [18–23]. Inhibition of Rev1 results in enhanced sensitivity and reduced mutation frequencies in response to DNA-damaging agents, such as UV light, hydrogen peroxide, cisplatin, and X-rays [24–28].

Mutations in human *REV1* have been detected in a minority of tumors [29], and single-nucleotide polymorphisms (SNPs) in the *hREV1* gene have been linked to various types of human cancer [30–32]. As with other TLS polymerases, upregulation of hREV1 is associated with the pathogenesis of human cancer [33]. Since hREV1 plays a critical role in TLS, and TLS contributes to the pathogenesis of tumors and to drug resistance by promoting tolerance of DNA damage, targeting hREV1 might be a promising approach for improving the outcome of chemotherapy. Recently, Wojtaszek J et al. reported that a small-molecule inhibitor of mutagenic translesion DNA synthesis (JH-RE-06), which disrupts the interaction between hREV1 and/or REV7, sensitizes cancer cells to cisplatin in vitro and in vivo [34].

Currently, despite the fact that several *Rev1*-mutated mouse models have been established, there is very little data regarding the role of Rev1 dysregulations in carcinogenesis in vivo (Table 1). Here we introduce four complementary Rev1 mutated mouse models, including the *Rev1* Tg mouse model generated in our laboratory, and we discuss their distinctive advantages for carcinogenesis research. This is the review based on the authors’ presentation at the Open Symposium of the Japanese Environmental Mutagen Society (JEMS) in 2017 [35].

**Table 1:**
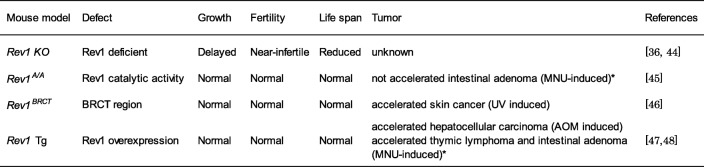
Mouse models of *Rev1* dysregulation

* Sasatani et al., manuscript in preparation

### *Rev1* KO mice

*Rev1* knockout (KO) mice were first generated with the objective of investigating the role of Rev1 in somatic hypermutation (SHM) [36]. The authors reported that *Rev1* KO mice showed delayed growth, a shortened life-span and were nearly infertile [36]. It should be noted that in addition to SHM, Rev1 is also involved in class switch DNA recombination (CSR), and both SHM and CSR are critical for maturation of the antibody response [37–39]. SHM and CSR are initiated by activation-induced cytidine deaminase (AID), which deaminates deoxycytosine (dC) residues to yield deoxyuridine (dU): deoxyguanine (dG) mispairs. These mispairs then trigger DNA repair processes facilitated by Uracil DNA glycosylase (Ung), Mismatch repair (MMR) proteins, and Rev1 [40]. SHM analysis demonstrated that mutation frequency and distribution were similar in B-cells from *Rev1* KO and wild type mice [36]. In contrast, the mutation spectra were significantly altered by the deletion of *Rev1.* An almost complete absence of C to G transversions was observed in *Rev1* KO cells, accompanied by a moderate decrease in G to C transversions and an increase in A to T substitutions similar to Ung deficiency. Since the induction of A to T mutations is highly dependent on Pol η, it is possible that the Rev1 defect results in increased access of Pol η to sites of DNA lesions. It has been reported that G: C to C: G transversions during SHM are generated downstream of two pathways: Ung2-dependent/Msh2-independent and Ung2/Msh2-dependent pathways [41]. Rev1 is indispensable in the former pathway and it plays the main role in the latter pathway, although in this case it can be replaced by other TLS polymerases. During CSR Rev1 recruits Ung to switch (S) regions and enhances dU glycosylation [42]. Nevertheless, Rev1 deficiency only slightly reduces CSR. In contrast, double Rev1/Msh2 defects lead to ablation of CSR, similarly to double Ung/Msh2 deficiency. CSR analysis has revealed that Rev1 exerts its functions in CSR via its non-catalytic properties.

Mouse embryonic fibroblasts (MEF) derived from *Rev1* KO mice have been reported to proliferate more poorly than wild-type cells [43]. Similarly, *Rev1* KO hematopoietic stem cells display competitive and proliferative disadvantage [44]. Furthermore, the additional disruption of *Xpc,* which is essential for global-genome nucleotide excision repair (ggNER), results in progressive loss of bone marrow, and fatal aplastic anemia between 3 and 4 months of age [44]. This finding suggests that Rev1-dependent TLS protects the genomic and functional integrity of the hematopoietic system in coordination with ggNER. In summary, the currently existing data is limited to genome instability and mutagenesis, but carcinogenesis studies on *Rev1* KO mice have not been published, and the impact of *Rev1* deficiency on cancer development in vivo remains unknown.

### Rev1^AA^ mice

**Fig. 1:**
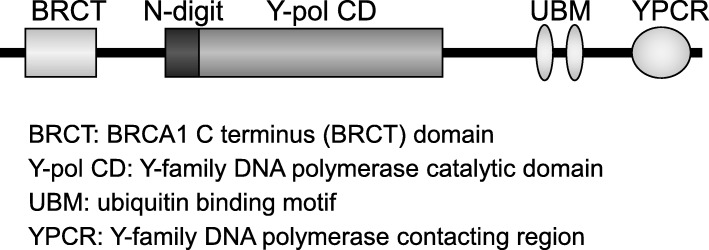
Structure of Rev1

*Rev1*^*AA*^ mice are defective specifically for *Rev1* catalytic activity due to mutations in a Y-family DNA polymerase catalytic domain of Rev1 (Fig. 1) [45]. *Rev1*^*AA*^ mice develop normally and are fertile. SHM analysis has demonstrated that B-cells from *Rev1*^*AA*^ mice are characterized by reduced overall mutation frequency and decreased mutagenesis at both G:C and A:T base pairs. This contrasts the abovementioned increase in A to T substitutions in *Rev1* KO mice, and one likely explanation is that *Rev1*^*AA*^ might inhibit the access of Pol η to sites of DNA lesions by remaining at the abasic site. Carcinogenesis study conducted in our laboratory suggests that *Rev1*^*AA*^ does not affect chemically-induced mutagenesis and carcinogenesis (Sasatani et al., manuscript in preparation). Thus, the catalytic domain of *Rev1* appears to be dispensable for either normal development or tumorigenesis.

### Rev1 ^BRCA1 C terminus (BRCT)^ mice

*Rev1*^*BRCT*^ mice carry a deletion in the BRCT domain of *Rev1*, which, however, retains catalytic function (Fig. 1) [46]. *Rev1*^*BRCT*^ exhibit a normal phenotype spontaneously, while being sensitive to exogenous DNA-damaging factors and exhibiting lower levels of ultraviolet C (UVC)-induced mutagenesis. Interestingly, despite the reduced mutagenesis, *Rev*^*BRCT*^;*Xpc* KO mice have been shown to be more vulnerable to skin carcinogenesis than *Xpc* KO mice. The authors concluded that this paradoxical phenotype was due to the induction of inflammatory hyperplasia that facilitates tumor promotion. Thus, the *Rev1*^*BRCT*^ mouse model is useful for interrogating the distinctive roles of *Rev1* in the initiation versus the promotion step of tumor development.

### *Rev1* Tg mice

*Rev1* Tg mice were generated in our laboratory by using the metallothionein promoter 1 (MT-1) to achieve inducible expression [47]. We found that the *Rev1* transgene was expressed at low levels in the liver and kidney, but at dramatically higher levels in the thymus, spleen, and lymph nodes. In order to determine whether overexpression of *Rev1* would influence spontaneous tumor initiation and progression, we monitored cohorts of Wt and *Rev1* Tg mice over their lifespan (> 2 years). No significant effect on overall survival or tumor incidence was observed, suggesting that overexpression of *Rev1* by itself is not sufficient to stimulate tumorigenesis. However, our study revealed that overexpression of *Rev1* promotes development of chemically-induced tumors, namely azoxymethane (AOM)-induced hepatocellular carcinoma, and *N*-methyl-*N*-nitrosourea (MNU)-induced thymic lymphoma and intestinal adenomas (Sasatani et al., manuscript in preparation) [47, 48]. Furthermore, in a comparative analysis of MNU-induced carcinogenesis in *Rev1* Tg (Homo) mice, which are homozygous (Tg+/Tg+) for the *Rev1* transgene, versus heterozygous *Rev1* Tg mice, we provided evidence that *Rev1* overexpression accelerates tumorigenesis in proportion to the *Rev1* expression level. Following MNU treatment, we observed enhanced mutagenesis and suppressed apoptosis in proportion to the level of *Rev1* overexpression.

**Fig. 2:**
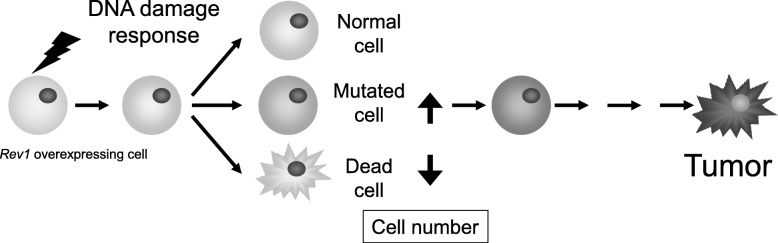
Model of accelerated chemically-induced tumorigenesis mediated by *Rev1* overexpression. Overexpressed *Rev1* suppresses apoptosis and increases the mutation frequency after the treatment of chemical reagents. The surviving fraction of mutated cells was higher under *Rev1* overexpression, resulting in acceleration of carcinogenesis

Our data implies that overexpression of *Rev1* promotes mutagenic TLS to safeguard replication on damaged templates, consequently inhibiting apoptosis and accelerating tumorigenesis (Fig. 2) (Table 2). Although the role of *REV1* overexpression in human carcinogenesis remains poorly understood, human cells overexpressing *REV1* exhibit a comparable to the abovementioned phenotype, with enhanced mutation frequency and hindered cell death, therefore, it can be clearly stated that regulation of Rev1 levels is required for maintaining genomic stability and tumor suppression (Table 2) [47].

**Table 2:**
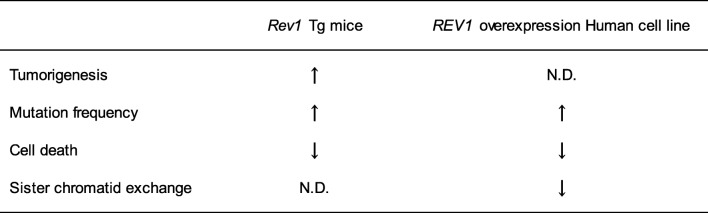
Summarized our data from *Rev1* Tg mice and human HT1080 cell line with a Tet-ON system in which human REV1

*ND* not detected

## Conclusions

Rev1 is a member of the TLS polymerase family and plays a key role in this mutagenic pathway, which allows the bypass of modified DNA bases and respectively, facilitates proliferation even in the presence of extensive DNA damage, such as during chemotherapy. Therefore, inhibition of the TLS pathway may be a promising strategy to tackle the problem of resistance to chemotherapy and to improve the therapeutic outcome.

Here we have introduced several mouse models with disruptions in *Rev1* functions, including the *Rev1* Tg mouse model generated in our laboratory. Recently we have reported that in *Rev1* Tg mice the elevated *Rev1* expression allows cells with mutations to survive after DNA damages, resulting in an acceleration of tumorigenesis [47, 48]. However, in vivo data like this is almost completely absent from the literature. We hope that data from studies employing *Rev1*-mutated mouse models will soon become available and will help us elucidate the mechanisms of *Rev1*-mediated tumorigenesis and chemotherapy resistance, so that we can in the future harness the therapeutic potential of TLS targeting.

## Data Availability

Not applicable.

## Abbreviations

Rev1: Reversionless 1
SHM: Somatic hypermutation
Tg: Transgenic
TLS: Translesion synthesis
